# The imaging quantification of multiple organs by dynamic ^18^F-FDG PET/CT in discharged COVID-19 patients: A prospective pilot study

**DOI:** 10.7150/ijms.73801

**Published:** 2022-09-06

**Authors:** Jijin Yao, Jing Liu, Lei Bi, Yiying Huang, Lu Wang, Fanwei Zhang, Ying Wang, Hongjun Jin

**Affiliations:** 1 Guangdong Provincial Key Laboratory of Biomedical Imaging, Fifth Affiliated Hospital, Sun Yat-sen University, Zhuhai, Guangdong Province 519000, China.; 2 Department of Head and Neck Oncology, Cancer Center, Fifth Affiliated Hospital, Sun Yat-sen University, Zhuhai, Guangdong Province 519000, China.; 3 Department of Respiratory Medicine, Fifth Affiliated Hospital, Sun Yat-sen University, Zhuhai, Guangdong Province 519000, China.; 4 Department of Information Technology, Fifth Affiliated Hospital, Sun Yat-sen University, Zhuhai, Guangdong Province 519000, China.; 5 Department of Nuclear Medicine, Fifth Affiliated Hospital, Sun Yat-sen University, Zhuhai, Guangdong Province 519000, China.

**Keywords:** COVID-19, ^18^F-FDG, Dynamic PET/CT, Patlak, Abnormal lesion

## Abstract

**Purpose:** To early identify abnormal lesions by applying the ^18^F-FDG PET dynamic modeling approach for discharged patients recovering from COVID-19.

**Methods:** Seven discharged COVID-19 patients (COVID-19 group), twelve healthy volunteers (control group 1), and eight cancer patients with normal pulmonary function (control group 2) were prospectively enrolled. Control group 1 completed static ^18^F-FDG PET/CT only; COVID-19 group and control group 2 completed 60-min dynamic ^18^F-FDG PET/CT. Among COVID-19 group and control group 2, the uptake of FDG on the last frame (at 55-60 min) of dynamic scans was used for static analysis. Prior to performing scans, COVID-19 patients provided negative real-time Reverse Transcription-Polymerase Chain Reaction (rRT-PCR) of SARS-CoV-2, normal lung functions test, and normal laboratory test. Organ-to-liver standard uptake ratio (OLR, i.e. SUV_max_^evaluated organ^/ SUV_max_^liver^) from conventional static data and Patlak analysis based on the dynamic modeling to calculate the ^18^F-FDG net uptake rate constant (K_i_) were performed.

**Results:** Compared to the control groups, COVID-19 patients at two to three months after discharge still maintained significantly higher K_i_ values in multiple organs (including lung, bone marrow, lymph nodes, myocardium and liver), although results for regular OLR measurements were normal for all discharged COVID-19 patients. Taking the image of lung as an example, the differences of SUV_max_ images between COVID-19 group and control group were hard to distinguish. In contrast, a high ^18^F-FDG signal of the lung among the COVID-19 group was observed for K_i_ images.

**Conclusion:** The K_i_ from ^18^F-FDG PET/CT dynamic imaging quantification might contribute to identifying residual lesions for COVID-19 survivors.

**Trial registration:** The trial is registered with ClinicalTrials.gov, number NCT04519255 (IRB-approved number, K52-1).

## Introduction

The coronavirus disease 2019 (COVID-19) pandemic, caused by severe acute respiratory syndrome coronavirus 2 (SARS-CoV-2), has resulted in over 600 million confirmed cases with over 6 million deaths globally as of August 23^th^, 2022. During this period, more than 570 million COVID-19 patients have been discharged from hospitals worldwide. Several studies have described the long-time health consequences of patients that recovered from COVID-19 1, 2; however, reliable imaging method to quantify underlying lesions for individuals that need early intervention remains not well understood. Therefore, exploring an imaging method for early identification of underlying lesions among discharged COVID-19 patients is of public health importance.

The ^18^F-FDG PET/CT has a high sensitivity for revealing abnormal metabolic lesions utilizing a non-invasive approach 3. This method has also been suggested to be important in detecting and monitoring inflammatory diseases 4, 5. In addition to estimating the inflammation of lungs, ^18^F-FDG PET/CT is beneficial for assessing metabolic and functional states for various organs throughout the body. For example, in Chefer's MERS-CoV animal model, increased bone marrow uptake over long periods were observed in follow-up ^18^F-FDG PET/CT 6. Therefore, ^18^F-FDG PET/CT has the potential to reveal underlying lesions in discharged COVID-19 patients. In previous studies, the maximum standardized uptake values (SUV_max_) have been commonly utilized for PET imaging quantification 7, 8. Nevertheless, factors like body mass index (BMI), uptake kinetics, or post-injection time may influence SUV outcomes 9. The absolute quantized Patlak kinetic model was used to calculate the ^18^F-FDG net uptake rate constant (K_i_) from linear modeling of graphical data 10.

To fill current gaps in knowledge, we applied the ^18^F-FDG PET/CT dynamic modeling method, Patlak analysis, to assess imaging quantification for multiple organs among discharged COVID-19 patients. Additionally, two control groups were included to evaluate metabolic activity for each organ. The present study may provide a new direction for early identification of underlying lesions among discharged COVID-19 patients.

## Methods

### Participants

From February 5^th^ to February 12^th^, 2020, patients discharged with COVID-19 (COVID-19 group) at our hospital were prospectively enrolled for this study. Eligible participants included: (a) diagnosis and treatment protocol for COVID-19 consistent with the World Health Organization (WHO) interim guidance 11; (b) performing 60-min dynamic PET/CT scans two to three months following discharge; (c) negative real-time Reverse Transcription-Polymerase Chain Reaction (rRT-PCR) of SARS-CoV-2 within three days prior to performing PET/CT scans; (d) normal laboratory test and pulmonary function test before performing PET/CT scans; (e) no history of diabetes or chronic respiratory diseases (e.g., asthma, bronchiectasis, and chronic bronchitis); and (f) complete clinical records. Overall, the COVID-19 group included seven patients that met the inclusion criteria to be enrolled in the analysis, including four cases of severe pneumonia and three of mild pneumonia. Twelve healthy volunteers (no documented record of SARS-CoV-2 infection or tumor history) who completed static PET/CT only were classified as control group 1. Another eight patients with non-metastatic, newly diagnosed thyroid cancer who completed 60-min dynamic PET/CT scans before anti-tumor therapy (e.g., surgery, radiotherapy, chemotherapy, immunotherapy, and targeted therapies) were classified as control group 2. The Research Ethics Committee of Sun Yat-sen University Cancer Center approved the study (IRB-approved number, K52-1), and written informed consent was obtained from all participants.

### ^18^F-FDG PET/CT assessment

Patients were asked to fast for a minimum of six hours prior to PET/CT scans. PET/CT imaging was conducted via a 112-ring digital light guide PET/CT (uMI780, United Imaging, China) according to published guidelines for PET/CT imaging 12. Helical CT was implemented from the head to proximal thigh prior to PET procurement based on the standardized protocol. Static acquisitions from head to proximal thigh were obtained 45 minutes post ^18^F-FDG injection and lasted for a period of 15 minutes. Dynamic acquisitions were done as follows: imaging started at the time of injection of ^18^F-FDG (3.75 MBq/kg produced from Guangzhou Atom High Tech Radiopharmaceutical Co.,Ltd., Guandong Province, China) and continued for 60 minutes. Dynamic scans were conducted from the lung apex to the liver for the COVID-19 group and control group 2. Each dynamic PET study lasted 60 minutes that contained 48 frames (time × frame: 5s × 18, 10s × 6, 30s × 5, 60s × 5, 150s × 8, 300s × 6) and was adjusted for isotope decay, scattering events, and random coincidence.

### PET data acquisition and analysis

The ^18^F-FDG uptake of myocardium, spinal cord, lung, bone marrow, lymph nodes, and liver were evaluated for the three study groups. To obtain the time activity curve (TAC) for the evaluated organs, ellipsoid volumes of interest (VOIs) were drawn over manually for each organ. The regions of SUV_max_ for myocardium and spinal cord were sketched as VOIs for each organ. Regarding lung, only those with suspected pulmonary abnormal lesions (i.e., patchy shadows, ground-glass opacity, and consolidated nodule) that both radiologists have confirmed, were sketched in COVID-19 group, and the same site was drawn in the control group 2. To determine whether the bone marrow has high metabolic uptake, the regions of SUV_max_ in the sternum and rib were delineated as VOIs. The vertebrae were not included because the free fluorine-18 tends to accumulate in the vertebrae and affects the results. The visible (≥3 mm) mediastinal lymph nodes, para-aortic lymph nodes, and hilar lymph nodes were delineated as VOIs. If no visible lymph nodes were observed, the regions of SUV_max_ in the corresponding lymph node station were delineated. The liver dome area is susceptible to motion artifact resulting from breathing, the area 3-5 cm below the upper edge of the right lobe of the liver was delineated as VOIs.

**Figure 1:**
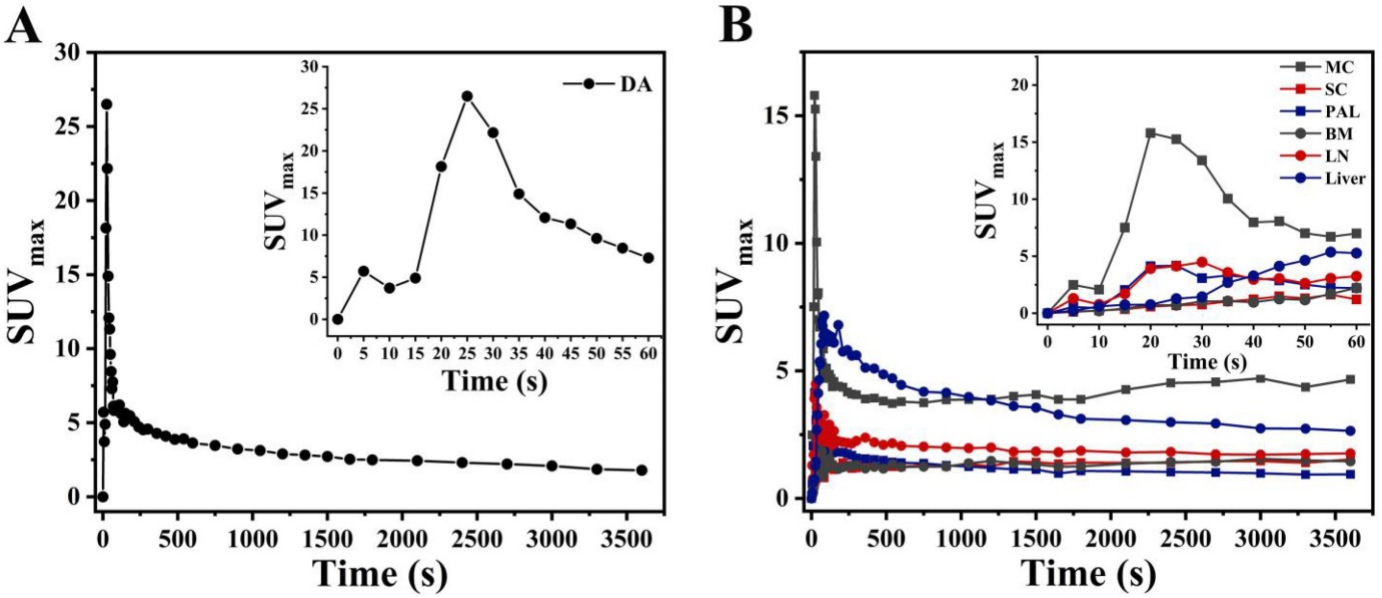
** PET image-derived input function from a dynamic acquisition.** Input function (the first 60s after injection) on descending aorta (A). Time activity curves (TAC) of myocardium (MC), spinal cord (SC), pulmonary abnormal lesions (PAL), bone marrow (BM), lymph nodes (LN), and liver from COVID-19 group.

Carimas software (http://turkupetcentre.fi/carimas/) published and freely distributed by the Turku PET Center was used for data and image analysis. The plugin of “Parametric image filter” from Carimas was used to generate static (SUV) and dynamic parametric (K_i_) PET images. **Figure S1** described the detail steps to generate parametric image. Conversely, VOIs were drawn manually on early frames in descending aorta (DA) to obtain the image-derived input functions (IDIF) in each patient. To minimize spillover and partial volume reactions, regions were small and far from the myocardial wall 13. These input VOIs were then projected onto all 48 frames yielding whole arterial blood TAC, (i.e., IDIF) (**Figure 1A**). The VOIs of evaluated organs were then projected on descending aorta given the dynamic study to provide TACs (**Figure 1B**). Overall, dynamic analyses for the COVID-19 group and control group 2 were performed on organs within the range single field of view. Detailed methods of the dynamic analysis were described in detail in our previous study 14. Additionally, the SUV_max_ of evaluated organs were determined. Among COVID-19 group and control group 2, the uptake of FDG on the last frame (at 55-60 min) of dynamic scans were used for static analysis. To reduce variation of experiments, i.e., possible inaccuracies regarding uptake kinetics, post-injection time, and BMI 15, the organ-to-liver standard uptake ratio (OLR, i.e. SUV_max_^evaluated organ^/SUV_max_^liver^) of evaluated organs was consequently calculated.

### Laboratory test and pulmonary function test

Methods for laboratory confirmation of COVID-19 were previously described in detail 16. Blood was collected from all patients to determine the presence of D-Dimer, C-reactive protein (CRP), lactate dehydrogenase (LDH), and lymphocyte (LY). Laboratory tests were performed every day during the first week after admission, and once every 1-2 days thereafter or adjusted frequency of laboratory test according to patient's medical condition. We performed lung function tests following the National Thoracic Society and European Respiratory Society (ATS-ERS) guidelines 17. The parameters measured including forced expiratory volume in the first second (FEV1), forced vital capacity (FVC), forced expired flow at 50% of FVC (FEF_50_), total lung capacity (TLC), residual volume (RV), carbon monoxide diffusing-capacity (DLCO), and carbon monoxide diffusing-capacity corrected for alveolar volume (DLCO/VA). All lung function values were reported as a percentage for the predicted values after adjusting for age, sex, weight, and height. The lung function test was required to be completed one month after discharge. If the test was abnormal, reexamination of lung function test is required the next month.

### Statistical analysis

The Statistical Package for the Social Sciences 19.0 was utilized for all analysis (Chicago, IL, USA). Clinical and demographic characteristics were obtained for each study cohort reported. Regional group differences in OLR and K_i_ values were compared between COVID-19 and the other two control groups using one-way non-parametric ANOVA (Kruskal-Wallis) test. A *p*-value < 0.05 was the threshold for statistical significance.

## Results

### Baseline clinical characteristics of subjects

For the COVID-19 group, the mean time and standard deviation (SD) from illness onset to admission was 5.9 (4.6) days, and the mean time to discharge was 23.7 (5.8) days. **Figure S2** presents how the examinations were performed and treatments were managed for each COVID-19 patient during the study period (i.e., given as the time from the illness onset to the day of PET/CT examination). A total of 367 laboratory tests were performed during the study period (i.e., given as the time from the illness onset to the day of last follow up). A total of 40 imaging examinations were performed, most of which were concentrated within 1-2 weeks after admission. The mean time (SD) of using antibiotic, antivirotic, anti-inflammatory, and passive immunization were 14.3 days (3.6), 9.3 days (3.8), 8.4 days (2.6), and 4.1 days (1.9) respectively. Baseline demographic characteristics were compared among COVID-19 group, control group 1, and control group 2. There were no significant differences regarding the male-female ratio, BMI, age at PET scan, and years of education among the study groups (*p* >0.05 for all; **Table 1**). In addition, chronic respiratory diseases (e.g., asthma, bronchiectasis, and chronic bronchitis) were not reported among all included patients.

### Lung function recovery and laboratory recovery

At admission, the abnormal laboratory test of D-Dimer, CRP, LDH, and LY counts were observed in 4 patients (4/7), 7 patients (7/7), 5 patients (5/7), and 5 patients (5/7), respectively. **Figure S3** presents the dynamic changes of laboratory indicators across pneumonia types. Laboratory indicators tended to increase in the first week after admission, and then gradually return to normal. The mean time from admission to full recovery in laboratory test was 12.8 days (ranging 10-15 days). At one month after discharge, only one patient (Case 1) suffered a slightly impaired lung function (i.e., FVC [75.7%], DLCO [68.0%], and DLCO/VA [76.0%]) (**Table 2**). However, abnormal lung function disappeared at two months following discharge for this patient (**Table S1**).

### Pulmonary inflammation abnormal lesions

**Figure 2:**
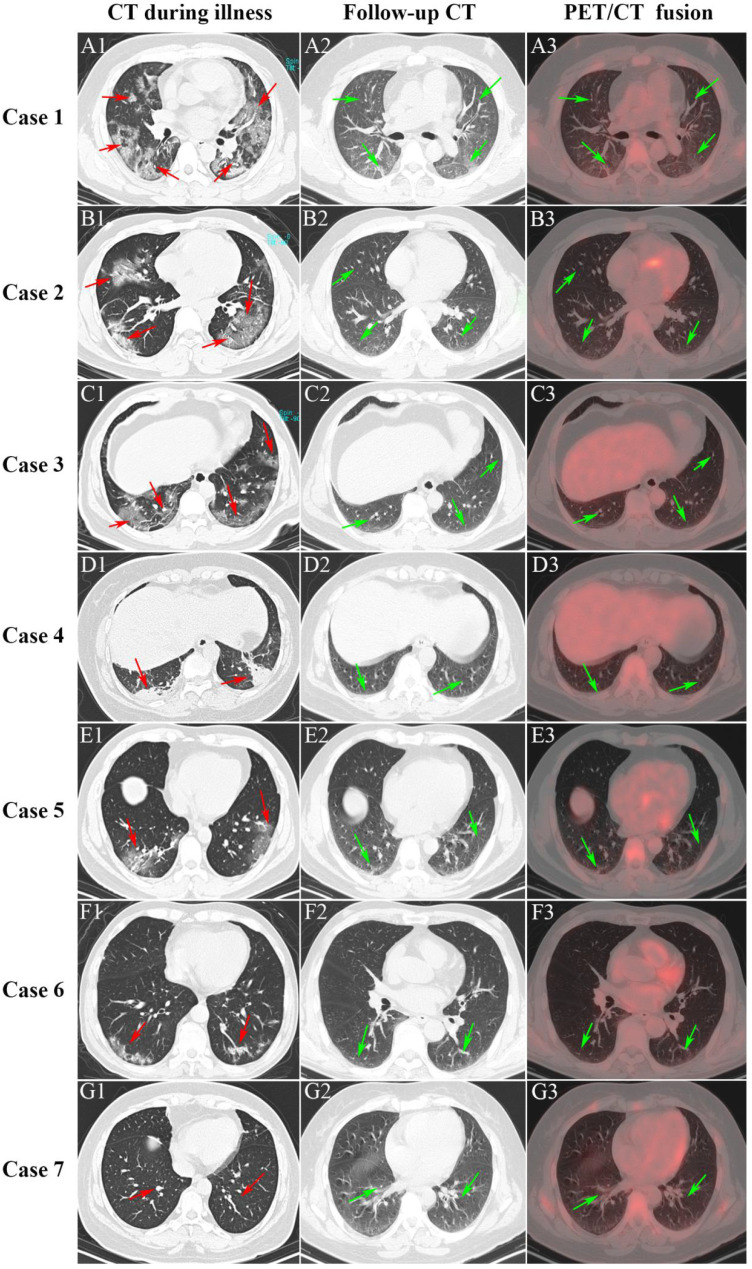
** Serial CT and PET/CT demonstrated recovery of COVID-19**. The first four patients (Case 1-4) suffered severe pneumonia and the last three patients (Case 5-7) suffered mild pneumonia.

For the COVID-19 group, the mean (SD) time from discharge to the date of static ^18^F-FDG PET/CT examination was 59.0 (12.6) days. **Figure 2** presents the CT imaging manifestation during illness (vertical: A1-G1), follow-up CT (vertical: A2-G2), and PET/CT (vertical: A3-G3) among seven COVID-19 survivors. Of the seven patients, the first four patients (Case 1-4) suffered severe pneumonia and the last three patients (Case 5-7) suffered mild pneumonia. Case 1 showed multiple patchy and light consolidation with patchy shadows in both lungs during illness (A1; red arrows). Follow-up CT showed obvious absorption of lesions in both lungs (A2; green arrows), however, increased ^18^F-FDG uptake was hard to observe in both lungs. The other three severe pneumonia (Case 2-4) groups presented patchy ground-glass opacities in both lungs with patchy consolidation lesions in it (B1-D1; red arrows) during illness. The interstitial pneumonia of both lungs receded greatly in the follow-up CT, and only a small amount of grid-like thickening of interlobular septa can be seen (B2-D2; green arrows). Moreover, no increase of ^18^F-FDG uptake was observed (C2-D2; green arrow) among Case 2 to 4. The last three patients with mild pneumonia (Case 5-7) showed scattered ground-glass opacities during illness (E1-G1; red arrows). Only scattered interlobular septum thickenings in the follow-up CT (E2-G2; green arrow) was present, and increased ^18^F-FDG uptake was not observed among the mild pneumonia (E3-G3; green arrows).

### Analysis of OLR values and K_i_ values among different groups

**Figure 3:**
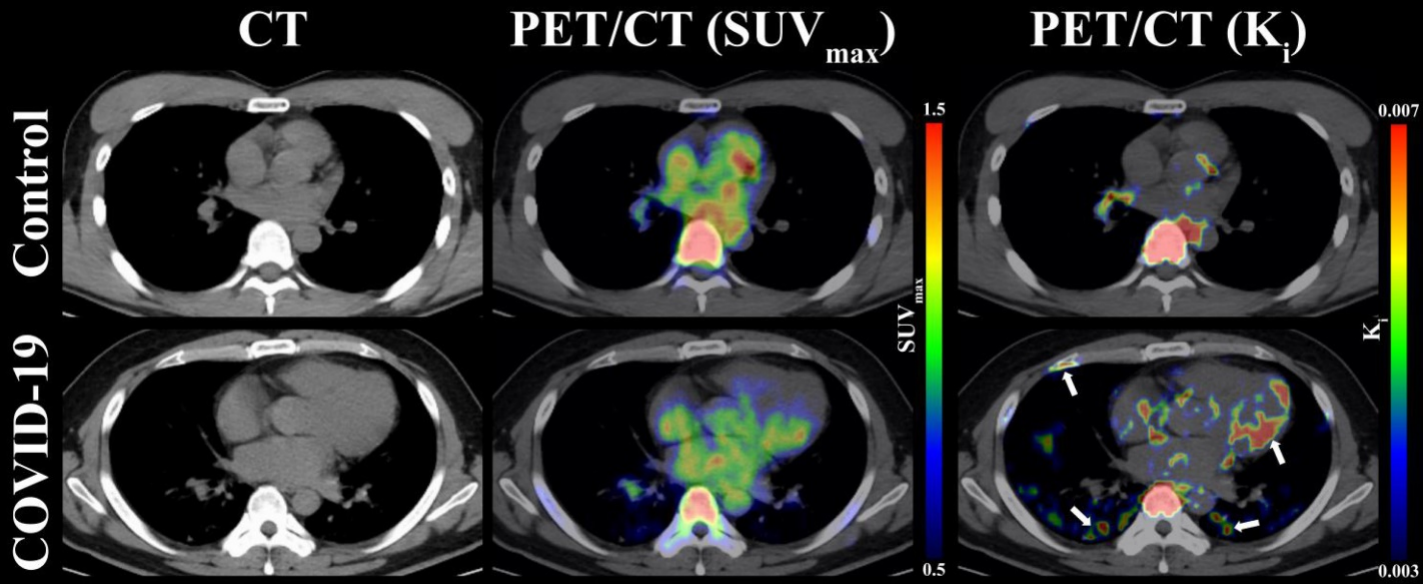
** Representative images of lung, myocardium, and bone marrow under CT, PET(SUV_max_)/CT, and PET(K_i_)/CT between COVID-19 group and control group 2.** The white arrows indicate multiple abnormal lesions for COVID-19 patients at two to three months following discharge.

As presented in **Table 1**, the OLR values were compared from five organs (i.e., lung, bone marrow, lymph nodes, myocardium, and spinal cord) among COVID-19 group, control group 1, and control group 2. Compared to control group 1 and control group 2, COVID-19 group presented with insignificant difference OLR in all evaluated organs (*p* > 0.05 for all, **Table 1**). Additionally, the values of OLR between control group 1 and control group 2 were comparable in all five organs (*p* > 0.05 for all, **Table 1**). Consistent with the result of OLR analysis, the K_i_ values of spinal cord (0.0069 ± 0.0010 min^-1^ vs. 0.0069 ± 0.0013 min^-1^; *p* = 0.969) were comparable between COVID-19 group and control group 2. However, significantly higher K_i_ values in lung (*p* < 0.001), bone marrow (*p* = 0.002), lymph nodes (*p* < 0.001), myocardium (*p* = 0.002), and liver (*p* = 0.009) were observed in COVID-19 group compared with control 2 group (**Table 1**). Representative PET/CT images of lung and myocardium are illustrated in **Figure 3**. The differences of evaluated organs between COVID-19 group and control group 2 were hard to distinguish. There was slightly an increased ^18^F-FDG uptake in both posterior lung fields (white arrow) on SUV_max_ images of discharged COVID19 patients, which are not evident among the control group 2. In contrast, a high ^18^F-FDG signal of lung and myocardium (white arrow) from the COVID-19 group can be observed in K_i_ images.

## Discussion

To the best of our knowledge, this is the first study to utilize dynamic ^18^F-FDG PET/CT for early identification of abnormal lesions among discharged patients recovering from COVID-19. Compared to the control groups, COVID-19 patients at two to three months after discharge still maintained significantly higher K_i_ values in multiple organs (lung, bone marrow, lymph nodes, liver, and myocardium), although results for regular SUV_max_ measurements, along with lung functions and laboratory tests were normal for all COVID-19 discharged patients. This cross-sectional study suggests that K_i_ from ^18^F-FDG PET/CT dynamic imaging quantification may provide more sensitive noninvasive method in detection of abnormal lesions among COVID-19 patients.

Recently, a follow-up for COVID-19 survivors indicated that up to 63% of patients had persistent fatigue or muscle weakness for roughly six months on average after COVID-19 2. Unfortunately, effective drugs to relieve the above symptoms are lacking. There are potentially multifactorial underlying mechanisms for sustained COVID-19 symptoms and may include direct effects from bone marrow abnormalities the immunological response, and corticosteroid therapy 18-20. In our study, we observed that K_i_ values of bone marrow from the COVID-19 group were significantly higher than K_i_ values for the control group. Consistent with the current study, Chefer et al. identified amplified ^18^F-FDG uptake in the bone marrow post MERS-CoV infection 6. Additionally, Dietz et al. 21 reported increased bone marrow uptake in COVID-19 patients as well. However, we could not deduce whether increased bone marrow uptake is directly related to chronic fatigue or muscle weakness. Hence, it is necessary to further assess the relationship between bone marrow abnormality and chronic fatigue by combining the results of bone marrow interlacement for COVID-19 survivors.

Literature has documented that ^18^F-FDG PET/CT acts as an important evaluator for inflammatory and infectious pulmonary diseases, such as COVID-19 22. Qin and colleagues 23 included four patients illustrating ^18^F-FDG PET/CT findings for individuals with acute respiratory disease produced from COVID-19 infection. They reported that three out of the four patients having multiple ^18^F-FDG-positive lymph nodes within the mediastinum and hilar region, and the lesions on ^18^F-FDG PET/CT regressed further after antiviral therapy. For SARS, autopsies suggest a large number of angiotensin 2 expression in lymph nodes and the spleen 24. The lesions of these immune organs may be due to direct attack of the virus and indirect immune injury 25. However, the evidence on the immune system recovery of COVID-19 survivors is minimal. In the current study, dynamic analysis observed lymph nodes in the COVID-19 group had statistically significant increases in K_i_ values than in the control group. This might indicate that the immune status of COVID-19 patients at two to three months after discharge is still unlike that of healthy people.

In our study, two to three months after discharge was selected as the imaging time point for PET/CT. This is due to the following two to three months after discharge is widely considered the appropriate time-point for initial assessment of recovery from pneumonia 26. Additionally, most pneumonia would have regressed or largely resolved. As for COVID-19 progress, COVID-19 disease may damage extra-pulmonary organs, such as kidneys, heart, and other organs 26-29. A previous study reported that COVID-19 could result in cardiac complications, including arrhythmia, heart failure, or myocardial infarction 29. Autopsies revealed the degeneration and focal necrosis in local cardiomyocytes 29, which was infiltrated with monocytes, lymphocytes, and neutrophils. However, it remains unclear whether COVID-19 recovered patients still have abnormal lesions in extra-pulmonary organs. Our results indicated significantly higher K_i_ values of the myocardium and liver in the COVID-19 group than control group 2. However, it is not possible to confirm whether there are residual lesions in the organs through histological confirmation for COVID-19 discharged patients. We therefore suggest that a consistent follow-up for those previously infected with COVID-19 is essential for understanding associations between extra-pulmonary diseases and SARS-CoV-2 infection.

Currently, SUV is widely used for quantitative evaluation of clinical ^18^F-FDG PET/CT examinations. However, the SUV methodology has obvious limitations, such as unsatisfactory test stability and uptake time dependence, which can adversely impact the reliability of the SUV 9, 30-31. Compared with SUV, OLR and Patlak analysis could obviously remove some of the SUV limitations 10, 32. We therefore selected OLR and Patlak analysis in the current study. On SUV images of discharged COVID19 patients, a slightly increased FDG uptake in both posterior lung fields could be seen, which indicated that an SUV image may also be helpful in the detection of the abnormal focus. But no significant difference of five evaluated organs, including lung, between COVID-19 group and other two control groups were observed according to SUV. In contrast, a high ^18^F-FDG signal of several evaluated organs from COVID-19 group can be observed in K_i_ images. Moreover, K_i_ values of lung, bone marrow, lymph nodes, liver and myocardium in the COVID-19 group were significantly higher than that in control group 2. This suggests K_i_ image provides more sensitive noninvasive method than SUV image in detection of abnormal lesions in discharged patients recovering from COVID-19.

In the current study, the significant difference of OLR in myocardium between COVID-19 group and other two control groups were not observed. These findings need to be interpreted with caution since image protocol for this study was not optimized to evaluate myocardium, which may affect the measurement of static uptake in myocardium. However, dynamic analysis was further conducted in our study, and the fasting time had little effect on the results of dynamic analysis 33. This study was unable to obtain dynamic PET/CT for healthy volunteers, and dynamic PET/CT patients with thyroid cancer were selected for the control group, potentially impacting the results. However, dynamic PET/CT was strictly restricted for our hospital, and it is only available to patients who have received approval from an ethics committee. To reduce the impact of thyroid cancer on the results of the study, the thyroid cancer patients adopted strict inclusion criteria. They were excluded for having distant metastases, regional lymphatic metastases, and other comorbidities. We must note that the normal pre-operative evaluation does not always warrant the non-metastatic thyroid cancer after surgery or even after ^131^I ablation. However, the static metabolic activity of evaluated organs was comparable between thyroid cancer patients and healthy volunteers, which contributed to reduce deviation within the study. Second, we were unable to collect data on pulmonary function test from two control groups since pulmonary function tests were not routinely performed in this population. Although pulmonary function tests were not performed for the control groups, patients from control groups had strict inclusion criteria, including previous fitness and no symptoms of shortness of breath, wheezing, or dyspnea. However, including pulmonary function tests would have been a more ideal measure of comparisons between patient and control groups. Finally, we failed to report the correlation analysis between the increasing K_i_ values and symptoms after discharge among COVID-19 survivors in the present study, due to failing to collect symptoms after discharge from COVID-19 patients. Further studies are needed to better understand this correlation.

In conclusion, we observed COVID-19 patients at two to three months following discharge continued to have a significantly higher K_i_ values for multiple organs, which may inspire novel concepts for recovery of COVID-19 survivors. Compared with the regular OLR, the K_i_ values were more sensitive in detection of abnormal lesions among COVID-19 patients. Despite the small sample size, future longitudinal assessments in larger, less selective cohorts may provide greater insight on the causal factors associated with the health consequences for discharged hospital patients recovering from COVID-19.

## Supplementary Material

Supplementary figures and tables.

## Figures and Tables

**Table 1:** Comparison of demographic characteristics, OLR, and K_i_ values among the studied groups

Characteristics	COVID-19	Control 1	Control 2	*P* value^a^		
				COVID-19 vs Control 1	COVID-19 vs Control 2	Control 1 vs Control 2
Participants, No	7	12	8	0.169	0.315	0.967
Men	5	4	3	N/A	N/A	N/A
Women	2	8	5	N/A	N/A	N/A
BMI, mean (SD), kg/m^2^	27.1 (2.9)	26.8 (2.7)	26.3 (2.4)	0.674	0.531	0.876
Age at PET scan, mean (SD), y	43.7 (10.2)	41.6 (8.7)	42.3 (9.3)	0.269	0.791	0.547
Years of education, mean (SD)	15.3 (2.8)	15.7 (2.3)	15.1 (2.5)	0.812	0.941	0.774
**OLR of evaluated organs^b^**						
lung	0.143±0.036	0.108±0.025	0.110±0.024	0.271	0.314	0.946
bone marrow	0.716±0.183	0.648±0.103	0.628±0.062	0.623	0.461	0.924
lymph nodes	0.485±0.117	0.439±0.072	0.436±0.105	0.649	0.546	0.954
myocardium	1.645±0.667	1.700±0.439	1.702±0.361	0.884	0.914	0.999
spinal cord	0.533±0.169	0.493±0.076	0.481±0.104	0.674	0.416	0.931
**K_i_ (min^-1^) values of evaluated organs^c^**						
lung	0.0028±0.0011	N/A	0.0009±0.0002	N/A	<0.001	N/A
bone marrow	0.0063±0.0024	N/A	0.0032±0.0003	N/A	0.002	N/A
lymph nodes	0.0066±0.0020	N/A	0.0029±0.0013	N/A	<0.001	N/A
myocardium	0.0240±0.0089	N/A	0.0105±0.0035	N/A	0.002	N/A
spinal cord	0.0069±0.0010	N/A	0.0069±0.0013	N/A	0.969	N/A
liver	0.0077±0.0026	N/A	0.0036±0.0005	N/A	0.009	N/A

Abbreviations: OLR, organ-to-liver standard uptake ratio; BMI, Body mass index; SD, standard deviation; N/A, not applicable; COVID-19, COVID-19 survivors who completed dynamic PET/CT at two to three months after discharge; Control 1, health volunteers who completed static PET/CT; Control 2, patients with non-metastatic, newly diagnosed thyroid cancer (T1-3N0M0, based on 8^th^ edition AJCC system) who completed both dynamic PET/CT scans before anti-tumor therapy. ^a^ *P* values are depicted for the group comparisons. ^b^ For static analysis, the activity concentration in evaluated organs was measured at 55-60 min after ^18^F-FDG injection. ^c 18^F-FDG PET dynamic modeling approach, Patlak analysis, was applied to investigate the imaging quantification of lung, bone marrow, and lymph nodes.

**Table 2:** Pulmonary function among COVID-19 patients one month following discharge

Parameter^#^	Severe (n = 4)				Mild (n = 3)		
	Case 1	Case 2	Case 3	Case 4	Case 5	Case 6	Case 7
**Spirometry**							
FVC%pred, (≥80% pred)	75.70^§^	83.00	127.90	134.90	84.60	101.20	102.70
FEV1%pred, (≥80% pred)	80.40	83.20	117.30	128.60	83.60	93.60	94.90
FEF50%pred, (≥65%pred)	113.10	75.50	90.90	90.50	87.30	73.60	68.50
**Diffusion capacity**							
DLCO%pred, (≥80%pred)	68.00^§^	82.80	81.20	119.60	92.50	81.20	80.20
DLCO/VA%pred, (≥80%pred)	76.00^§^	89.60	80.2	114.30	112.80	84.90	86.50
**Lung volume**							
TLC%pred, (≥80%pred)	81.80	81.40	114.80	117.00	80.70	90.50	93.70
RV%pred, (≥65%pred)	104.90	77.50	93.00	102.20	74.20	96.30	78.60

Values are presented as mean±standard deviation (SD). FVC, forced vital capacity; FEV1, forced expiratory volume in the first second; FEF_50_, forced expired flow at 50% of FVC; DLCO, carbon monoxide diffusing-capacity; DLCO/VA, carbon monoxide diffusing-capacity corrected for alveolar volume; TLC, total lung capacity; RV, residual volume. ^#^ The normal range of pulmonary parameter in each organ is indicated in parentheses. ^§^ The pulmonary parameter of the organ is lower than that of the normal interval.
