# Supplementary material for: The imaging quantification of multiple organs by dynamic 18F-FDG PET/CT in discharged COVID-19 patients: A prospective pilot study
Source: Int J Med Sci. 2022 Sep 6;19(10):1539–47. doi: 10.7150/ijms.73801 (PMC9515694; doi:10.7150/ijms.73801)
Supplement: Supplementary file 1 — Supplementary figures and tables. [file ijmsv19p1539s1.pdf]

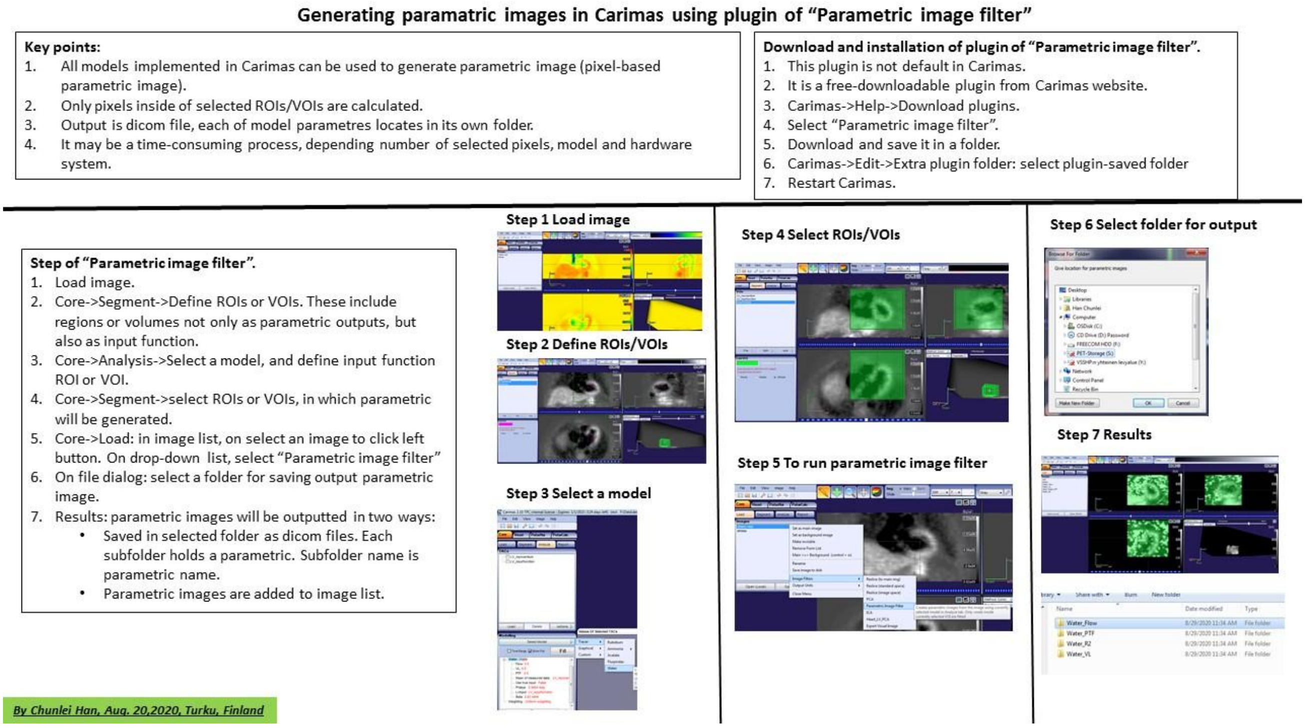

Figure S1. Generating parametric images in Carimas using plugin of “Parametric image filter”.

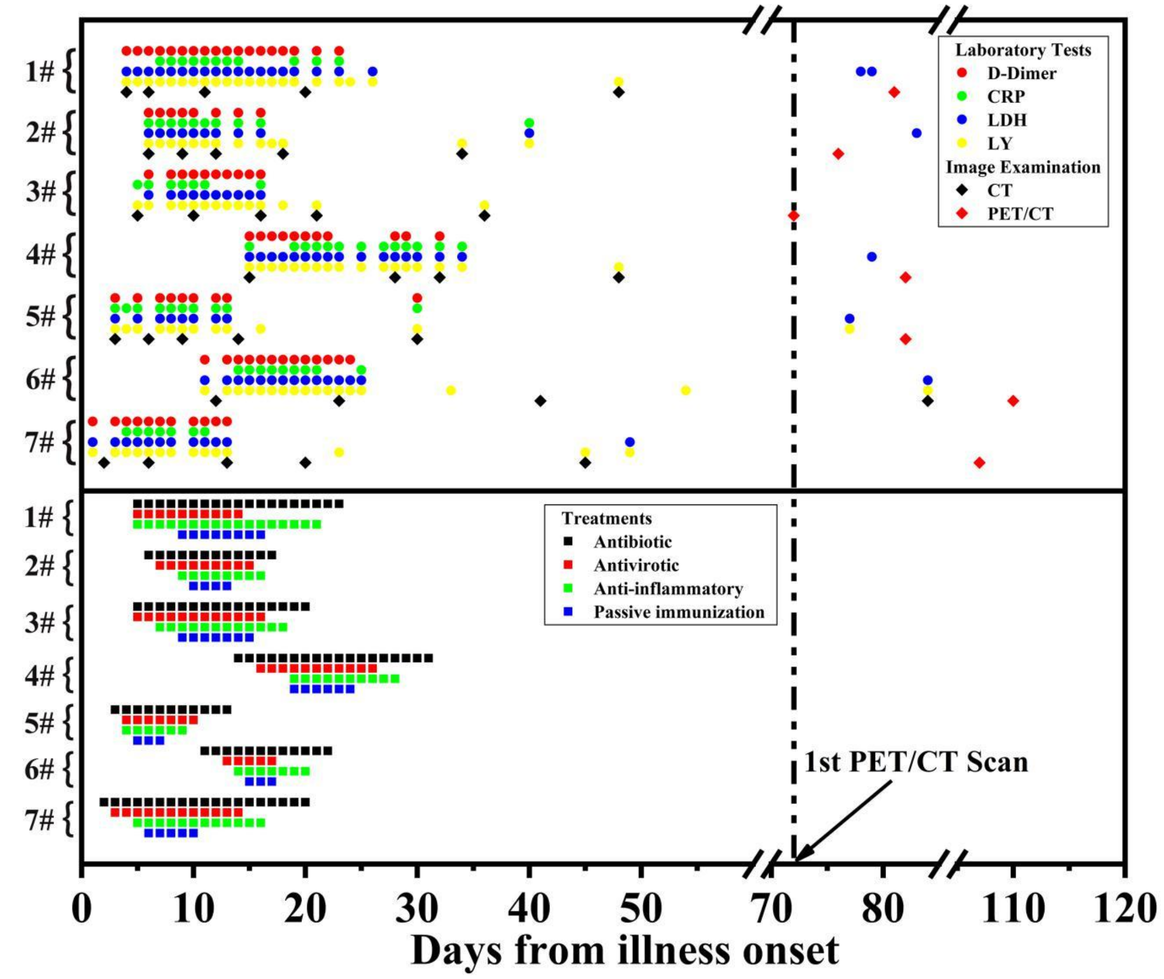

Figure S2. presents how the examinations were performed, and treatments were managed for each COVID-19 patient during the study period (e.g., given as time from illness onset to the day of last follow up). The dashed line indicates when the first PET/CT was performed. CRP, C-reactive protein; LDH, lactate dehydrogenase; LY, lymphocyte.

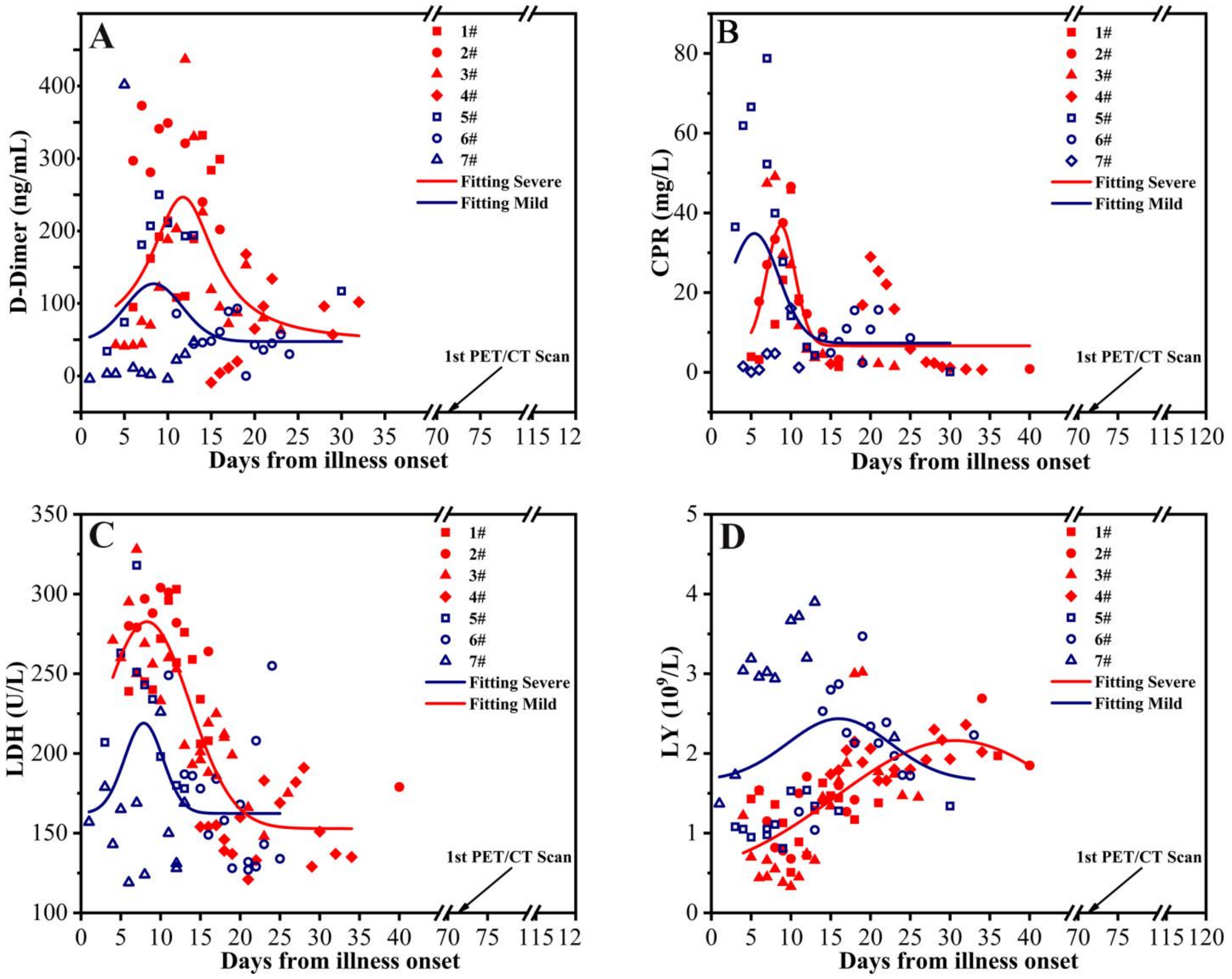

Figure S3. Laboratory tests indicates the full recovery states when the PET/CT were performed. (A). Among severe COVID-19 patients, D-Dimer increased notably and peaked 9–11 days after illness onset, followed by a rapid decreasing trend. Similar patterns were observed among mild COVID-19 patients within the first week after illness onset. However, the peak D-Dimer for mild was lower than that of severe COVID-19 and began to decline 3-4 days in advance. (B). CRP increased rapidly after illness onset among severe and mild COVID-19, and peaked at the 9th and 5th days, respectively. From there, declined rapidly and returned to normal range within 13-15 days. (C). Among mild COVID-19 patients, LDH increased in the first week after illness onset, and downward thereafter. Compared with mild COVID-19, the peak of LDH tended to occur at the illness onset for patients with severe COVID-19, followed by a long-term continuously decreasing trend. (D). LY increased rapidly after illness onset among severe and mild COVID-19, and peaked at 15-17 days and 29-31 days, respectively.

Table S1. Results of pulmonary function test at three months after discharge for the Case 1 patient.

Case 3	Spirometry			Diffusion capacity		Lung volume	
Parameter[#]	FVC%pred	FEV1%pred	FEF50%pred	DLCO%pred	DLCO/VA%pred	TLC%pred	RV%pred
	≥80% pred	≥80% pred	≥65%pred	≥80%pred	≥80%pred	≥80%pred	≥65%pred
	86.1	88.20	106.40	95.3	127.4	83..40	70.50

Values are presented as mean±standard deviation (SD). FVC, forced vital capacity; FEV1, forced expiratory volume in the first second; FEF_{50}, forced expired flow at 50% of FVC; DLCO, carbon monoxide diffusing-capacity; DLCO/VA, carbon monoxide diffusing-capacity corrected for alveolar volume; TLC, total lung capacity; RV, residual volume.

[#] The normal range of pulmonary parameter in each organ is indicated in parentheses.
